# Electrophysiological Characterization of Regular and Burst Firing Pyramidal Neurons of the Dorsal Subiculum in an Angelman Syndrome Mouse Model

**DOI:** 10.3389/fncel.2021.670998

**Published:** 2021-08-26

**Authors:** Prudhvi Raj Rayi, Hanoch Kaphzan

**Affiliations:** Sagol Department of Neurobiology, The Integrated Brain and Behavior Research Center, University of Haifa, Haifa, Israel

**Keywords:** Angelman syndrome, patch-clamp electrophsyiology, hippocampus, burst firing neurons, dorsal subiculum, mouse model

## Abstract

Angelman syndrome (AS) is a debilitating neurogenetic disorder characterized by severe developmental delay, speech impairment, gait ataxia, sleep disturbances, epilepsy, and a unique behavioral phenotype. AS is caused by a microdeletion or mutation in the maternal 15q11-q13 chromosome region containing *UBE3A* gene. The hippocampus is one of the important brain regions affected in AS mice leading to substantial hippocampal-dependent cognitive and behavioral deficits. Recent studies have suggested an abnormal increase in the α1-Na/K-ATPase (α1-NaKA) in AS mice as the precipitating factor leading to the hippocampal deficits. A subsequent study showed that the hippocampal-dependent behavioral deficits occur as a result of altered calcium (Ca^+2^) dynamics in the CA1 pyramidal neurons (PNs) caused by the elevated α1-NaKA expression levels in the AS mice. Nonetheless, a causal link between hippocampal deficits and major behavioral phenotypes in AS is still obscure. Subiculum, a region adjacent to the hippocampal CA1 is the major output source of the hippocampus and plays an important role in the transfer of information from the CA1 region to the cortical areas. However, in spite of the robust hippocampal deficits and several known electrophysiological alterations in multiple brain regions in AS mice, the neuronal properties of the subicular neurons were never investigated in these mice. Additionally, subicular function is also implied in many neuropsychiatric disorders such as autism, schizophrenia, Alzheimer’s disease, and epilepsy that share some common features with AS. Therefore, given the importance of the subiculum in these neuropsychiatric disorders and the altered electrophysiological properties of the hippocampal CA1 PNs projecting to the subiculum, we sought to examine the subicular PNs. We performed whole-cell recordings from dorsal subiculum of both WT and AS mice and found three distinct populations of PNs based on their ability to fire bursts or single action potentials following somatic current injection: strong bursting, weak bursting, and regular firing neurons. We found no overall differences in the distribution of these different subicular PN populations among AS and WT controls. However, the different cell types showed distinct alterations in their intrinsic membrane properties. Further, none of these populations were altered in their excitatory synaptic properties. Altogether, our study characterized the different subtypes of PNs in the subicular region of an AS mouse model.

## Introduction

Angelman syndrome (AS) is a rare neurogenetic disorder caused by the deletion or loss of function of the maternal *UBE3A* gene present on chromosome 15q11-q13 region (Magenis et al., 1987; Kishino et al., 1997; Matsuura et al., 1997). Normally, the paternal *UBE3A* gene, though present during the embryonic development is fully silenced in neurons 1 week after birth and from thereon has no substantial effect (Glenn et al., 1997; Judson et al., 2014; Sonzogni et al., 2020). AS is characterized by severe cognitive impairment, sleep disturbances, speech impairment, epilepsy, motor deficits, and a typical behavioral profile (Clayton-Smith and Laan, 2003; Williams et al., 2006; Bindels-de Heus et al., 2019). Prior studies in AS model mice have reliably replicated many of the clinical features of AS patients (Jiang et al., 1998; Miura et al., 2002; Silva-Santos et al., 2015). These clinical features, which manifest as cognitive and behavioral deficits in AS mice coincided with a plethora of electrophysiological abnormalities in various cortical and subcortical regions. Previous data in AS mice reported alterations in layer 2/3 pyramidal neurons (PNs) of the primary visual cortex (Wallace et al., 2012; Judson et al., 2016), layer 5 PNs of the medial prefrontal cortex (Rotaru et al., 2018), hippocampal CA1 PNs (Jiang et al., 1998; Kaphzan et al., 2011; Rayi et al., 2019, 2020), principal neurons of medial nucleus of the trapezoid body (Wang et al., 2018), striatal medium spiny neurons (Hayrapetyan et al., 2014), and cerebellar granule cells (Bruinsma et al., 2015). However, the neuronal properties of subicular PNs in AS model mice were never investigated. This is especially interesting given that subiculum acts as the major output structure of the hippocampus (Witter et al., 1989; Naber et al., 2000), and hippocampus is one of the most extensively studied brain regions in AS model mice (Jiang et al., 1998; van Woerden et al., 2007; Kaphzan et al., 2011, 2012, 2013). The subiculum receives converging inputs primarily from the CA1 region and the entorhinal cortex (Amaral, 1993; O’Mara et al., 2001). Earlier studies in the subiculum have reported three distinct subtypes of PNs classified as strong bursting (SB), weak bursting (WB), and regular firing (RF) neurons based on their response to a depolarizing current pulse (Staff et al., 2000; Jung et al., 2001). However, some studies have classified the subicular PNs into two functional groups: bursting and non-bursting (regular spiking) neurons (Mason, 1993; Stewart and Wong, 1993; Taube, 1993; Greene and Totterdell, 1997; Stewart, 1997; Dunn et al., 2018). An important characteristic of the subicular neurons is their ability to burst intrinsically at high frequencies upon suprathreshold current injection. Additionally, these high-frequency bursts of action potentials (APs) are functionally relevant in enhancing the fidelity of the information transfer between the neurons and fine-tuning the memory processing such as memory retrieval and spatial encoding (Sharp and Green, 1994; Gabrieli et al., 1997; Lisman, 1997; Izhikevich et al., 2003; Simonnet and Brecht, 2019). Furthermore, subiculum is affected in neurological disorders such as schizophrenia (Lieberman et al., 2018; Nakahara et al., 2018), Alzheimer’s disease (Hyman et al., 1990; Carlesimo et al., 2015; Lindberg et al., 2017), and epilepsy (Stafstrom, 2005), which share some common features with AS. Considering the anatomical positioning and the connectivity of the subiculum, together with its prominent role in neuropsychiatric disorders, we aimed to carry out a comparative study of the electrophysiological properties of different subicular PNs in AS mice and their WT littermates.

Using whole-cell patch clamp recordings in an *ex vivo* acute hippocampal slice preparation, we investigated the intrinsic membrane properties and the excitatory post-synaptic currents in the PNs of dorsal subiculum of AS mice. Consistent with previous studies, we found three types of neurons (SB, WB, and RF) in the AS subiculum distinguished by their firing patterns in response to depolarizing somatic current injection steps. The majority of neurons in this region were found to be bursting in nature. However, we did not observe any significant differences in the distribution of the different populations of subicular PNs between AS and WT mice. Further, we analyzed and compared these three distinct PN populations and found that only SB and RF neurons have reduced excitability whereas WB neurons have unaltered firing frequency in AS mice. Moreover, all the cell populations in AS mice showed distinct alterations in some of their intrinsic membrane properties with no changes in the extrinsic synaptic properties. Altogether, we outlined and characterized the different neuronal populations present in the subicular region of an AS mouse model.

## Materials and Methods

### Animals

In the present study, we used adult (2–3 m.o.) male, C57BL/6 AS mice and their WT littermates. Mice were group housed with access to water and food *ad libitum* and maintained on a 12 h light/dark cycle. AS model mice containing *Ube3a* maternal deletion (m^–^/p^+^) were generated by crossing female *Ube3a* breeder mice (m^+^/p^–^) with male WT (m^+^/p^+^) mice. A total of 26 animals (WT = 13, AS = 13) were used in our study.

### Ethics Approval

All the animal experiments were approved by the University of Haifa Institutional Ethics Committee.

### Hippocampal Slice Preparation

Mice were deeply anesthetized with a mixture of ketamine (10%, 200 mg/kg, Vetmarket, Israel) and xylazine (10%, 20 mg/kg, Vetmarket, Israel), and perfused transcardially with aerated, ice-cold cutting solution, containing (in mM): 110 sucrose, 60 NaCl, 3 KCl, 1.25 NaH_2_PO_4_, 28 NaHCO_3_, 0.5 CaCl_2_, 7 MgCl_2_, and 5 D-glucose. After decapitation, the brains were rapidly removed, and hippocampal coronal sections of 300 μm thickness were prepared using an SMZ7000 vibratome (Campden Instruments, United Kingdom). Throughout the dissection, brains were maintained in oxygenated ice-cold cutting solution. Following dissection, the hippocampal slices containing dorsal subiculum were transferred to a holding chamber with warm artificial cerebrospinal fluid (aCSF, maintained at 34°C) containing (in mM): 125 NaCl, 2.5 KCl, 1.25 NaH_2_PO_4_, 25 NaHCO_3_, 25 D-glucose, 2 CaCl_2_.2H_2_O, and 1 MgCl_2_.6H_2_O. After 30 min recovery in warm aCSF, the slices were stored at room temperature (RT, 24 ± 1°C) for a minimum of 1 h for further recovery. Individual slices were then transferred to a submerged recording chamber for performing whole-cell recordings from subicular PNs. All the solutions were aerated with 95% O_2_/5% CO_2_ mixture throughout the various steps of dissection, incubation, and recording. We acquired the PNs from coronal slices containing dorsal subiculum from regions −2.6 to −3.3 relative to bregma, using Allen brain atlas as reference (images 80–86).

### Whole-Cell Recordings of Subicular PNs

**FIGURE 1:**
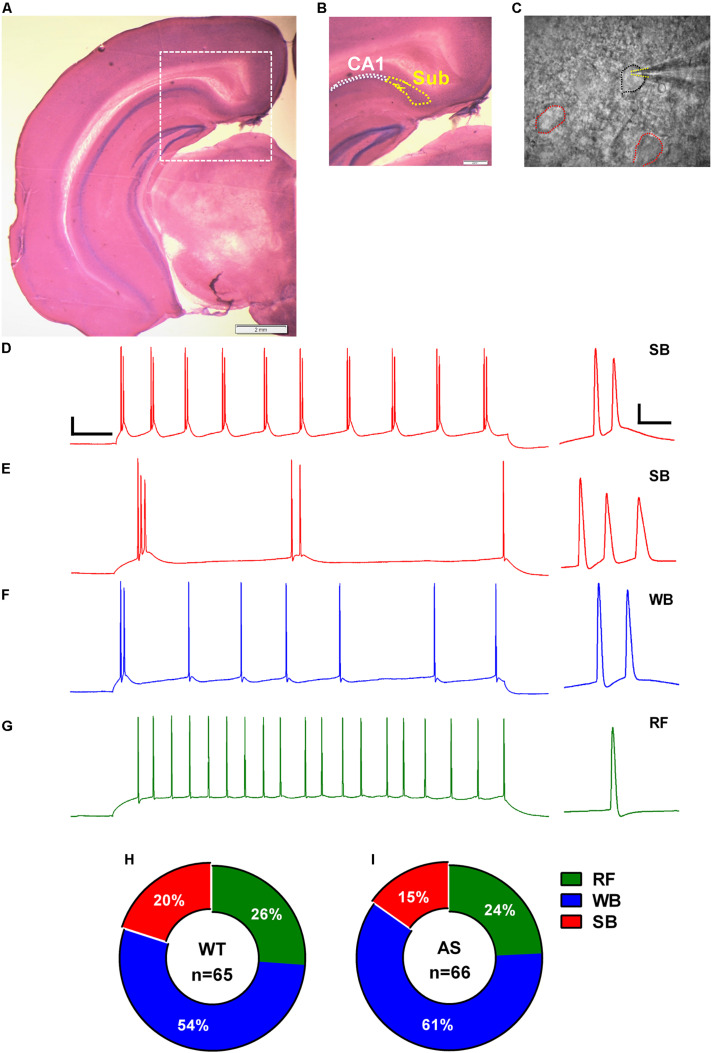
Identification, classification, and distribution of subicular pyramidal neurons. **(A)** Nissl-stained coronal brain section after electrophysiological recording showing the different hippocampal regions. **(B)** Zoomed-in region from **(A)** (dashed white square) showing the hippocampal CA1 pyramidal layer demarcated with white dotted outline and the adjacent subicular region (Sub) defined with yellow dotted outline. **(C)** DIC image of the subicular region from **(B)** showing a patched pyramidal neuron (PN; black outline) and its neighboring cells (red outlines). Dashed yellow lines mark the patch pipette in whole-cell mode. Note that the pyramidal neurons (PNs) in subiculum are sparsely distributed. Scale: 2 mm. **(E–G)** Representative traces of various responses of subicular PNs to a 1-s somatic current injection revealed **(D)** strong bursting (SB) neurons with multiple (>3) bursts, **(E)** SB neurons with only 2–3 bursts, **(F)** weak bursting (WB) neurons with only one burst followed by a train of single spikes, and **(G)** regular firing (RF) neurons with trains of single action potentials (APs). Scale: 20 mV, 100 ms. Right: Expanded view of the first burst or AP from the corresponding trace. Scale: 20 mV, 10 ms. The APs in all the traces were obtained from WT mice elicited by a somatic depolarizing current injection of 150 pA. The summary indicative of distribution of the subicular neurons in **(H)** WT and **(I)** AS mice. No significant differences were observed in the distribution pattern of different neuronal populations in the subiculum of WT and AS mice [χ*^2^* = 0.04, *p* = 0.84 for RF, χ*^2^* = 0.16, *p* = 0.68 for WB, and χ*^2^* = 0.37, *p* = 0.54 for SB neurons in chi-square test].

We used infrared differential interference contrast (IR-DIC) microscopy to illuminate and visualize the PNs of the dorsal subiculum. These subicular PNs were distinguished from CA1 PNs based on their morphology and location. Subicular cells are generally larger and sparsely distributed in comparison to the densely packed CA1 PNs. Further, we chose subicular cells that are located in a region in continuation to the *stratum pyramidale* layer of the CA1. Borosilicate glass pipettes (1B150F-4, WPI) with a resistance of 3–5 MΩ were pulled (P-1000; Sutter Instruments, Navato, CA). We used K-gluconate-based intracellular solution containing (in mM): 120 K-gluconate, 10 HEPES, 1 MgCl_2_.6H_2_O, 0.2 EGTA, 2 Mg-ATP, 0.2 Na_3_-GTP, with osmolarity of 290 mOsm and pH 7.3 to record the intracellular properties and excitatory postsynaptic currents of the subicular cells. The seal was ruptured after the cells reached a resistance of >2 GΩ. After entering whole-cell mode, we waited for at least additional 5 min for the diffusion of the internal solution prior to making any recordings. Only cells with a clear pyramidal soma and smooth surface were chosen for recording. The intrinsic membrane properties were acquired in current-clamp mode, and the excitatory synaptic properties were acquired in voltage-clamp mode at RT. Membrane potentials were not corrected for liquid junction potential. Series resistance was monitored continuously during the recordings and neurons with series resistance >30 MΩ and/or resting membrane potential (RMP) > −60 mV were excluded from the analysis. Recordings were sampled at 50 kHz, amplified using a Multiclamp 700B amplifier, digitized by a Digidata 1440 apparatus (Molecular Devices, San Jose, CA), and filtered at 10 and 2 kHz for all current-clamp and voltage-clamp recordings, respectively. We analyzed the data off-line using Clampfit 10 software (Molecular Devices, San Jose, CA). For representative images, we performed Nissl staining using cresyl violet on slices taken from the electrophysiological recordings to illustrate the area of recording in the subiculum and the clear demarcation between the dense CA1 cell layer and the sparsely distributed cells of the subicular region (Figures 1A,B).

### Assessment of Intrinsic Membrane Properties

Analysis of intrinsic membrane properties was performed as previously described (Rayi et al., 2020). Briefly, we injected a series of square current pulses of 1 s duration ranging from hyperpolarizing current step of -150 pA to a maximum depolarizing current step of 300 pA in increments of 50 pA to study membrane excitability. The traces obtained from depolarizing steps of 50–300 pA were used to generate firing rate curves by determining the AP frequency evoked at each level of current injection. We further used these traces to differentiate between the regular and burst firing PNs of the subiculum. Input resistance was measured using the trace elicited by -100 pA hyperpolarizing current injection obtained from the above experiment. Input resistance was calculated by linearly fitting voltage change to the injected current. Resting membrane potential (RMP) was analyzed from *I* = 0 pA trace averaged over 100 ms. For studying the intrinsic membrane properties of the neurons, single APs were triggered by injecting square current pulses for 10 ms in 10 pA steps. The single AP with its peak closest to the 5 ms time-point from the start of current injection was used to analyze the various AP parameters such as AP threshold, AP half-width, AP amplitude, and rheobase. In burst firing neurons, this protocol often resulted in a burst containing 2 APs (Mason, 1993). However, we did not analyze the second AP resulted from these bursts. For defining the AP threshold potential, we considered the point on the AP trace where its first derivative (d*V*/d*t*) reached 30 V/s. AP amplitude was measured from the threshold to the peak of the AP. AP half-width was calculated as the AP duration at the half-maximal amplitude. Medium afterhyperpolarizing potential (mAHP) was recorded by injecting a current pulse of 3,000 pA at 50 Hz for 3 s as was previously described by us and others (Gulledge et al., 2013; Chakraborty et al., 2017; Lander et al., 2019). mAHP was measured from the baseline RMP (averaged over 100 ms before the start of the first current step) to the first negative voltage peak after the last spike. Inter-spike interval (ISI) ratio was analyzed as a ratio between the shortest ISI and the average ISI of the APs elicited in response to the 300 pA current injection (Jarsky et al., 2008). We used about 2–4 slices per mouse to record from the subicular PNs. A total of 131 PNs were recorded (WT = 65, AS = 66). Analyses were performed by an experimenter blinded to the genotype.

### Assessment of Spontaneous Excitatory Postsynaptic Currents (sEPSCs)

AMPA receptor–mediated sEPSCs were recorded in voltage-clamp mode for 2 min at a holding potential of −70 mV. The currents were filtered at 2 kHz using a low-pass filter. The data were analyzed off-line using template search option in Clampfit 10 (Molecular Devices). Events <6 and >100 pA were not considered for analysis. Parameters like average amplitude, average frequency, and cumulative distribution curves of amplitudes and inter-event intervals were analyzed. For comparison of the cumulative distributions, we binned every 10 ms for inter-event intervals (IEI) between 0 and 1,000 ms, and every 1 pA for amplitudes between 6 to 80 pA.

### Statistics

All the data were analyzed using GraphPad Prism 6th and 7th edition software (GraphPad Software, La Jolla, CA). Distribution differences in the neuronal populations were calculated using Chi-square test. The comparison of intrinsic excitability between different types of cells in AS and WT mice were analyzed using two-sided unpaired Student’s *t* test. For comparisons of firing rate curve between groups, we utilized RM two-way ANOVA with Bonferroni’s *post hoc* multiple comparison test. Cumulative distribution curves of sEPSC amplitudes and inter-event intervals were analyzed using Kolmogorov-Smirnov (KS) non-parametric test. For all tests, ^∗^*p* < 0.05 (two-sided) was considered significant. The individual values, each obtained from a single cell, are represented as aligned dot plots with bar graphs of mean ± standard error of the mean (SEM). The description of the number of recorded cells (n) and the specific statistical test used are mentioned in the figure legends. The experimenter blinded to the genotype performed all analyses.

## Results

### Identification and Electrophysiological Classification of Subicular PNs

We obtained coronal slices containing dorsal subiculum from regions −2.6 to −3.3 relative to bregma using Allen brain atlas as reference (images 80–86). The subicular area starts as an extension to the CA1 *stratum pyramidale* layer and is clearly demarcated from the laminar CA1 region by a sparsely distributed cell layer (Figures 1A–C). Whole-cell patch clamp recordings were obtained from 131 (WT = 65 and AS = 66) randomly chosen, smooth-surfaced subicular PNs. We classified the subicular neurons based on the firing response to a 1 s depolarizing somatic current injection (Figures 1D–G). Consistent with previous studies (Greene and Totterdell, 1997; Staff et al., 2000; Jung et al., 2001), we found three distinct populations of PNs in the subiculum which we classified as strong bursting (SB), weak bursting (WB), and regular firing (RF) based on the number of bursts or single APs elicited during the suprathreshold current injection. A burst was defined as two or more (up to 6) APs occurring at a high frequency (>100 Hz). SB neurons fired at least two bursts containing 2–4 APs per burst followed by single spikes (Figures 1D,E), while WB cells showed only one burst containing 2–6 APs which were followed by trains of repetitive single APs (Figure 1F). RF neurons elicited only repetitive single spikes in response to the current injection with a frequency of <100 Hz (Figure 1G). Population analysis revealed no significant differences in the distribution of these neuronal subtypes between AS and their WT littermates. Consistent with previous studies (Mason, 1993; Staff et al., 2000; Dunn et al., 2018), we found that majority of the subicular cells were bursting in nature in both AS (50/66 neurons) and WT (48/65 neurons) mice. Among the WT, we found 13 SB (20%), 35 WB (54%), and 17 RF (26%) neurons (Figure 1H). On the other hand, we found 10 SB (15%), 40 WB (61%), and 16 RF (24%) neurons in AS mice (Figure 1I).

### Intrinsic Membrane Properties of RF Neurons Are Partially Altered in AS Mice

**FIGURE 2:**
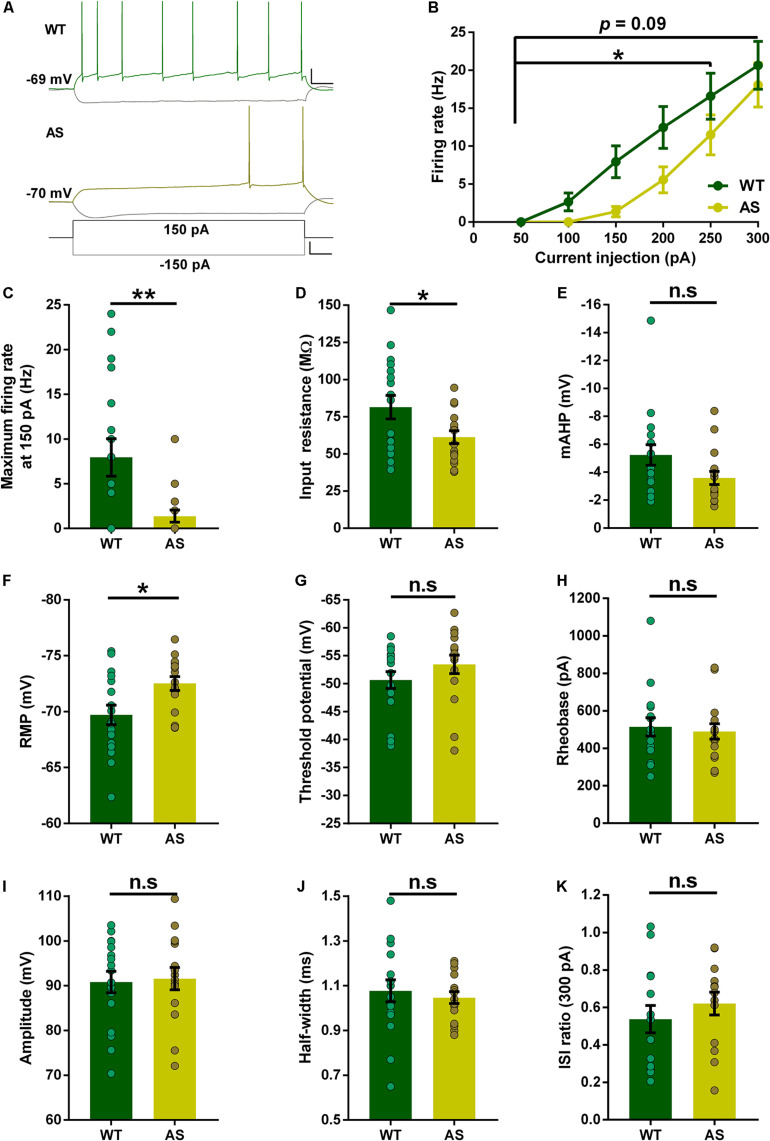
Partial alterations in the passive and active membrane properties of subicular RF PNs. **(A)** Representative traces of responses to a 150 pA depolarizing and –150 pA hyperpolarizing current injection in subicular RF neurons of WT (green), and AS (olive) mice. Scale: 20 mV, 100 ms. The bottom trace represents the step current used to elicit the above responses. Scale: 100 pA, 100 ms. **(B)** Firing frequency of all RF neurons in response to different somatic current injection steps ranging from 50 to 300 pA. The overall firing frequency of AS mice was significantly lower compared to their WT counterparts during 50–250 pA current injection [*F*_(__1_,_31__)_ = 4.50, *p* = 0.04 for main effect of genotype; *F*_(__4_,_124__)_ = 2.47, *p* = 0.04 for interaction of genotype and current injection in two-way RM ANOVA]. However, at higher current injection of 300 pA, the firing frequency remains unaltered in AS mice [*F*_(__1_,_31__)_ = 2.92, *p* = 0.09 for main effect of genotype; *F*_(__5_,_155__)_ = 1.82, *p* = 0.11 for interaction of genotype and current injection in two-way RM ANOVA]. **(C–K)** Aligned dot plots of passive and active membrane properties of RF neurons. **(C)** The average maximum firing rate at 150 pA current step is significantly lower in AS mice compared to their WT littermates [*t*_(__31__)_ = 2.90, *p* = 0.007] **(D)** Input resistance is significantly lower in AS mice compared to their WT controls [*t*_(__31__)_ = 2.19, *p* = 0.03]. **(E)** mAHP shows no difference between the genotypes [*t*_(__31__)_ = 1.85, *p* = 0.07]. **(F)** RMP is hyperpolarized in AS mice compared to their WT littermates [*t*_(__31__)_ = 2.60, *p* = 0.01]. **(G)** Threshold potential shows no alteration in AS and WT controls [*t*_(__31__)_ = 1.25, *p* = 0.22]. **(H)** The rheobase is unaffected between AS and WT mice [*t*_(__31__)_ = 0.39, *p* = 0.70]. **(I)** Amplitude is not altered between the genotypes [*t*_(__31__)_ = 0.22, *p* = 0.83]. **(J)** There is no significant difference in half-width between AS and WT mice [*t*_(__31__)_ = 0.53, *p* = 0.60]. **(K)** ISI ratio remains unaltered between the AS and WT littermates [*t*_(__26__)_ = 0.87, *p* = 0.39]. For ISI ratio: WT, *n* = 14 RF neurons, *N* = 10 mice; AS, *n* = 14 RF neurons, *N* = 7 mice. For the rest of the parameters: WT, *n* = 17 RF neurons, *N* = 10 mice; AS, *n* = 16 RF neurons, *N* = 7 mice. This variation in the cell numbers in ISI ratio analysis is because three neurons from WT group and two neurons from AS group fired no more than 2 APs at 300 pA current step, which made the calculation of ISI ratio unfeasible. Bar graphs of intrinsic membrane properties were analyzed by two-tailed unpaired Student’s *t* test. Data are represented as mean ± SEM and each data point represent a neuron. *p < 0.05; **p < 0.01; *n*.s, non-significant.

Previous studies have reported alterations in the intrinsic membrane properties of hippocampal CA1 PNs in AS mice (Kaphzan et al., 2011, 2013; Rayi et al., 2019, 2020). However, the PNs of the subiculum were never investigated in these mice. Using whole-cell recordings, we analyzed the resting membrane and firing properties in detail for each subset of subicular neurons in both WT and AS littermates. Overall, the RF neurons of AS mice (Figure 2A), exhibited a significant reduction in their excitability compared to the WT counterparts as shown in the relationship between injected currents and firing frequencies (*f–I* curves) (Figure 2B). However, this significant reduction between genotypes was only observed for medium current injections of up to 250 pA (Figures 2B,C and Supplementary Table 1). At higher current injection of 300 pA, these differences in the firing frequency between AS and WT mice are not significant (Figure 2B). Consistent with the *f*–*I* curve, we observed a significantly lower input resistance in the AS mice when compared to their WT littermates (Figure 2D). The mAHP of AS mice remained unaltered between the genotypes (Figure 2E). RMP in AS mice showed a significant hyperpolarization compared to their WT littermates (Figure 2F). Conversely, there were no differences in the threshold potential, rheobase, amplitude, half-width, and ISI ratio (Figures 2G–K) in RF PNs of AS mice compared to their WT controls.

### Intrinsic Properties of WB Neurons Remain Largely Unaltered in AS Mice

**FIGURE 3:**
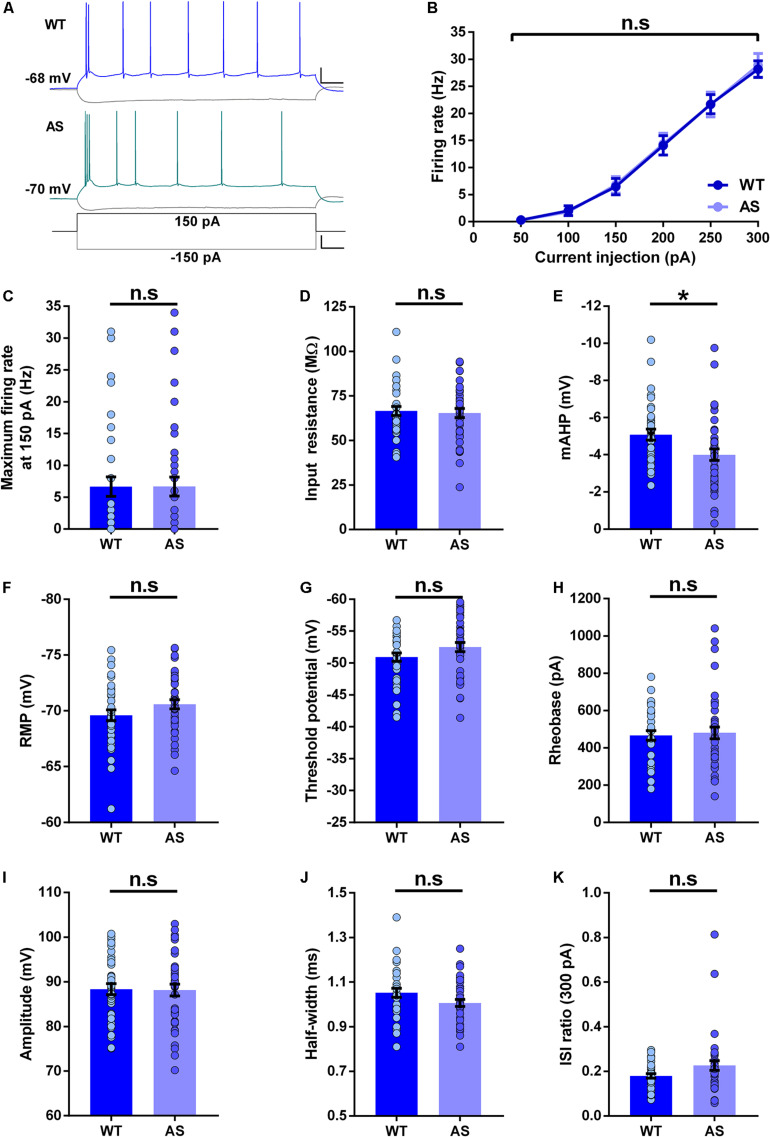
Passive and active membrane properties of subicular WB PNs. **(A)** Representative current-clamp recordings of WT (blue), and AS (teal) mice following a 150 pA depolarizing and –150 pA hyperpolarizing current injection in WB neurons of the subiculum. Scale: 20 mV, 100 ms. The bottom trace shows the step current used to elicit the above responses. Scale: 100 pA, 100 ms. **(B)** Summary of firing response curves of all WB PNs following somatic current injection steps ranging from 50 to 300 pA grouped according to the genotype. The firing frequency is unchanged in AS mice compared to the WT controls [*F*_(__1_,_73__)_ = 0.007, *p* = 0.93 for main effect of genotype; *F*_(__5_,_365__)_ = 0.06, *p* = 0.99 for interaction of genotype and current injection in two-way RM ANOVA]. **(C–K)** Bar graphs of passive and active membrane properties of WB neurons. **(C)** The average maximum firing rate remains unaltered between AS mice and their WT littermates at 150 pA current step [*t*_(__73__)_ = 0.008, *p* = 0.99]. **(D)** Input resistance is unchanged in AS mice in comparison to their WT counterparts [*t*_(__73__)_ = 0.30, *p* = 0.76]. **(E**) The mAHP is significantly lower in AS mice compared to the WT controls [*t*_(__73__)_ = 2.48, *p* = 0.01]. **(F)** RMP is not altered between the genotypes [*t*_(__73__)_ = 1.54, *p* = 0.13]. **(G)** Threshold potential remains unchanged between AS and WT controls [*t*_(__73__)_ = 1.60, *p* = 0.11]. **(H)** The rheobase is unaltered in AS mice compared to the WT mice [*t*_(__73__)_ = 0.34, *p* = 0.73]. **(I)** Amplitude is not different between the genotypes [*t*_(__73__)_ = 0.11, *p* = 0.91]. **(J)** Half-width is similar between AS mice and their WT littermates [*t*_(__73__)_ = 1.80, *p* = 0.08]. **(K)** ISI ratio shows no significant changes in the AS mice compared to the WT controls [*t*_(__72__)_ = 1.88, *p* = 0.06]. For ISI ratio: WT, *n* = 35 WB neurons, *N* = 10 mice; AS, *n* = 39 WB neurons, *N* = 13 mice. For the rest of the parameters: WT, *n* = 35 WB neurons, *N* = 10 mice; AS, *n* = 40 WB neurons, *N* = 13 mice. This variation in the cell number in ISI ratio analysis is because one neuron from the AS group fired not more than 2 APs at 300 pA, which made the calculation of ISI ratio unfeasible. Bar graphs of intrinsic membrane properties were analyzed by two-tailed unpaired Student’s *t* test. Data are represented as mean ± SEM and each data point represent a neuron. *p < 0.05, *n*.s, non-significant.

We next studied the intrinsic membrane properties of subicular WB neurons in AS mice and their WT littermates. The *f*–*I* curves of these neurons were not different between the AS and WT mice (Figures 3A,B). In addition, isolated comparisons of the firing frequencies at various current injection levels (100–250 pA) did not reveal any alterations in the excitability of these neurons (Figures 3B,C and Supplementary Table 2). Further, the input resistance of WB neurons remained unchanged in AS mice compared to their WT littermates (Figure 3D). However, the mAHP was significantly reduced in these neurons in AS mice (Figure 3E). We did not find any alterations in the RMP, threshold potential, rheobase, amplitude, half-width, and ISI ratio in the WB neurons of AS mice compared to their WT controls (Figures 3F–K).

### SB Neurons of the Subiculum Show Reduced Excitability in AS Mice

**FIGURE 4:**
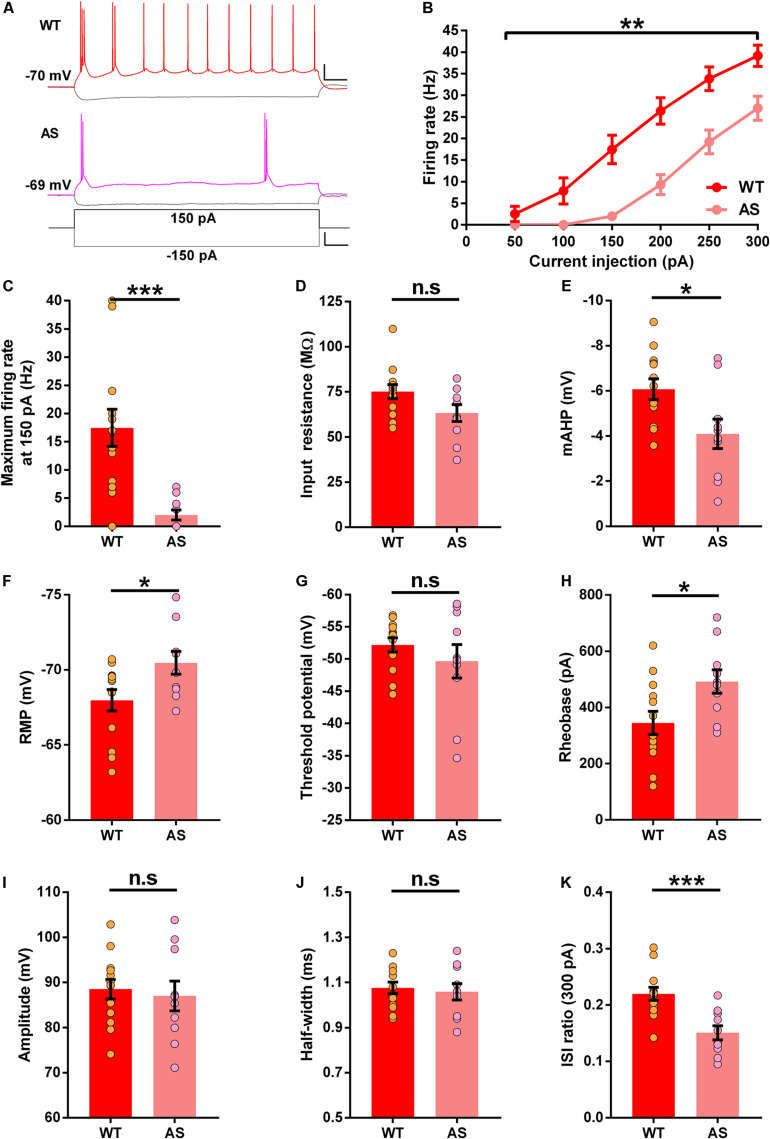
Altered excitability in the subicular SB PNs of AS mice. **(A)** Example recordings illustrating the response following injection of a 150 pA depolarizing and a –150 pA hyperpolarizing current step in SB neurons of WT (Red), and AS (Fuchsia) mice. Scale: 20 mV, 100 ms. The bottom trace represents the step current used to elicit the above responses. Scale: 100 pA, 100 ms. **(B)** Firing response curves of all SB PNs summarized according to the genotype and current injection steps ranging from 50 to 300 pA. AS mice exhibit a reduction in firing frequency compared to their WT counterparts [*F*_(__1_,_21__)_ = 13.13, *p* = 0.001 for main effect of genotype; *F*_(__5_,_105__)_ = 9.93, *p* < 0.0001 for interaction of genotype and current injection in two-way RM ANOVA; *post hoc* Bonferroni corrected comparison of current steps: *t*_(__126__)_ = 4.32, *p* = 0.0002, *t*_(__126__)_ = 4.77, *p* < 0.0001, *t*_(__126__)_ = 4.10, *p* = 0.0005, and *t*_(__126__)_ = 3.39, *p* = 0.005 for 150, 200, 250, and 300 pA respectively]. **(C–K)** Bar graphs with aligned dot plots representing the passive and active membrane properties of SB neurons. **(C)** The maximum firing rate at 150 pA current step is significantly lower in AS mice compared to their WT littermates [*t*_(__21__)_ = 4.00, *p* = 0.0006]. **(D)** Input resistance shows a downward trend in AS mice compared to their WT littermates [*t*_(__21__)_ = 1.96, *p* = 0.06]. **(E)** mAHP is significantly lower in AS mice compared to the WT controls [*t*_(__21__)_ = 2.54, *p* = 0.02]. **(F)** RMP in AS mice is significantly hyperpolarized compared to their WT littermates [*t*_(__21__)_ = 2.38, *p* = 0.03]. **(G)** Threshold potential remains unchanged between AS and WT littermates [*t*_(__21__)_ = 0.98, *p* = 0.34]. **(H)** The rheobase is significantly higher in AS mice when compared to their WT counterparts [*t*_(__21__)_ = 2.48, *p* = 0.02]. **(I)** Amplitude is similar between AS and WT controls [*t*_(__21__)_ = 0.40, *p* = 0.69]. **(J)** Half-width remains unaffected between the genotypes [*t*_(__21__)_ = 0.39, *p* = 0.69]. **(K)** ISI ratio is significantly reduced in AS mice compared to the WT controls [*t*_(__21__)_ = 3.98, *p* = 0.0007]. For all the parameters: WT, *n* = 13 SB neurons, *N* = 8 mice; AS, *n* = 10 SB neurons, *N* = 7 mice. Bar graphs of intrinsic membrane properties were analyzed by two-tailed unpaired Student’s *t* test. Data are represented as mean ± SEM and each data point represent a neuron. *p < 0.05; **p < 0.01; ***p < 0.001; *n*.s, non-significant.

The SB neuronal analysis between genotypes revealed multiple alterations in their intrinsic membrane properties. We observed a significant reduction in the firing rate of SB neurons in AS mice (Figures 4A,B). It is also worth noting that the firing rate of SB neurons in WT mice was higher compared to either RF or WB neurons (Supplementary Figure 1). The average firing rates between 100 and 250 pA current injections were significantly lower in AS mice than their WT counterparts (Figures 4B,C and Supplementary Table 3). However, we only found a trend toward a lower input resistance in the AS mice (Figure 4D). The mAHP of SB neurons in AS mice was significantly reduced (Figure 4E) and the RMP was significantly hyperpolarized in the SB neurons of these mice compared to their WT littermates (Figure 4F). We did not find any alterations in the threshold potential (Figure 4G). However, the rheobase was significantly higher in the SB neurons of AS mice compared to the WT controls (Figure 4H). The AP amplitude and half-width in these neurons remained unchanged between the genotypes (Figures 4I,J). Nevertheless, ISI ratio was significantly lower in the SB neurons of AS mice when compared to their WT littermates (Figure 4K).

### sEPSCs in Subicular PNs Are Mostly Unaltered in AS Mice

**FIGURE 5:**
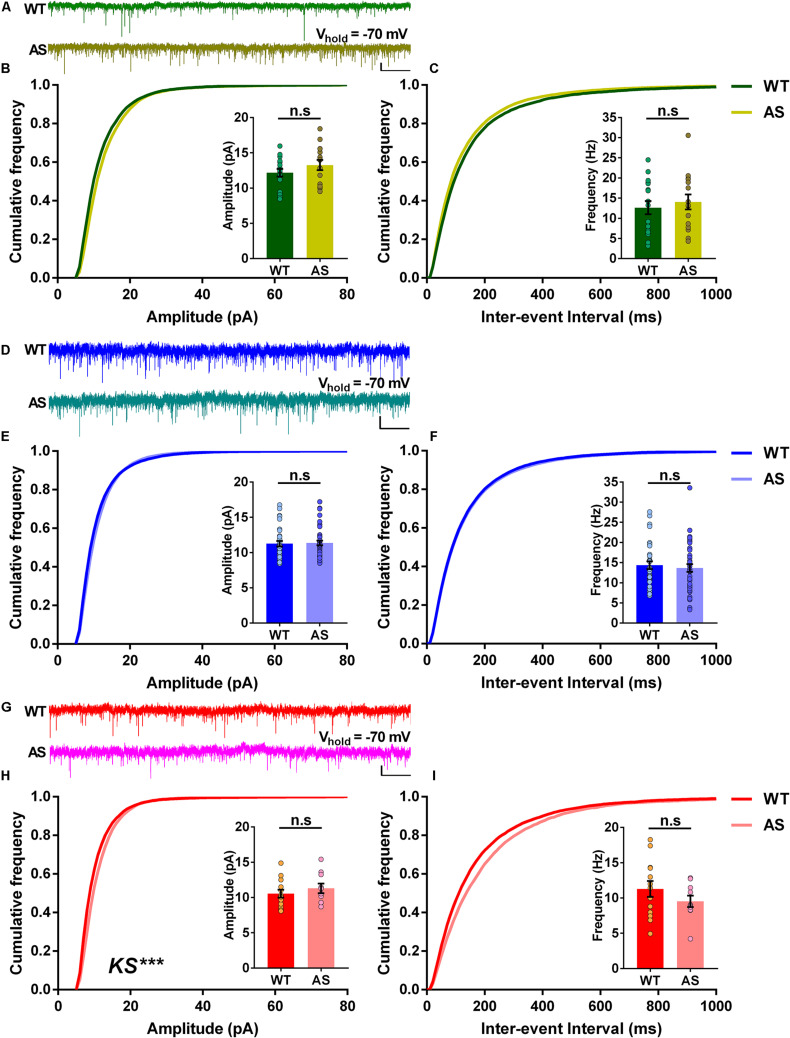
The spontaneous excitatory postsynaptic currents (sEPSCs) of different subicular PNs remain unaltered in AS mice. **(A)** Representative voltage-clamp traces of sEPSCs recorded from RF subicular PNs of WT (green) and AS (olive) mice. sEPSCs from different subicular populations were recorded by holding the cells at –70 mV. **(B)** Cumulative probability plots of sEPSC amplitudes and **(C)** inter-event intervals of the RF subicular PNs in WT and AS mice. The cumulative frequency distribution of AS mice shows no differences in amplitudes [KS test, *p* = 0.40] and inter-event intervals [KS test, *p* = 0.21] when compared to their WT controls. Insets show quantification of the average amplitude [*t*_(__30__)_ = 1.21, *p* = 0.23] and frequency of sEPSCs [*t*_(__30__)_ = 0.57, *p* = 0.57], which are not different between genotypes. For WT, *n* = 17 RF neurons, *N* = 10 mice; AS, *n* = 15 RF neurons, *N* = 7 mice. **(D)** Representative traces of sEPSCs recorded from WB subicular PNs of WT (blue) and AS (teal) mice. **(E)** Cumulative probability plots of sEPSC amplitudes and **(F)** inter-event intervals of the WB subicular PNs. The cumulative distribution plots of AS mice show similar amplitudes [KS test, *p* = 0.10] and inter-event intervals [KS test, *p* = 0.30] compared to their WT controls. Insets show bar graphs of mean amplitude [*t*_(__71__)_ = 0.19, *p* = 0.85] and frequency of sEPSCs [*t*_(__71__)_ = 0.50, *p* = 0.62], which did not differ between WT and AS mice. For WT, *n* = 33 WB neurons, *N* = 10 mice; AS, *n* = 40 WB neurons, *N* = 13 mice. **(G)** Representative traces of sEPSCs recorded from SB subicular PNs of WT (red) and AS (fucshia) mice. **(H)** Cumulative probability plots of sEPSC amplitudes and **(I)** inter-event intervals of SB subicular PNs. The cumulative plots of AS mice show higher amplitudes [KS test, *p* = 0.0003] with no differences in the inter-event intervals [KS test, *p* = 0.47] when compared to their WT controls. Insets show the quantification of average amplitude [*t*_(__21__)_ = 0.84, *p* = 0.41] and frequency of sEPSCs [*t*_(__21__)_ = 1.18, *p* = 0.25], which did not alter between the genotypes. For WT, *n* = 13 SB neurons, *N* = 8 mice; AS, *n* = 10 SB neurons, *N* = 7 mice. Average amplitude and frequency were analyzed using two-tailed unpaired Student’s *t* test. The data are represented as mean ± SEM and each data point represent a neuron. Cumulative distribution plots were analyzed using Kolmogorov-Smirnov non-parametric test. ***p < 0.001, *n*.s, non-significant. Scale: 10 pA, 2 s.

We further wanted to determine the incoming excitatory synaptic properties to the different neuronal populations of the subiculum. To this extent, we recorded sEPSCs in voltage-clamp mode, holding the cells at −70 mV. Analysis of the RF neurons revealed no significant differences in the cumulative frequency distributions of both amplitudes and inter-events interval in AS mice compared to the WT controls (Figures 5A–C). This coincided with the average amplitudes and average frequencies of sEPSCs, which were comparable in both genotypes (Insets, Figures 5B,C). Similarly, the WB neurons of AS mice showed no differences in the cumulative frequency distributions of amplitudes and inter-event intervals (Figures 5D–F), with similar average amplitudes and frequencies between AS and WT controls (Insets, Figures 5E,F). However, for SB neurons, we observed significant changes in the cumulative frequency distributions of their amplitudes but not their inter-event intervals in AS mice (Figures 5G–I). However, these changes did not result in significant differences in their corresponding average amplitudes and frequencies compared to the WT controls (Insets, Figures 5H,I). Overall, these results suggest that the loss of *Ube3a* did not significantly affect the excitatory inputs to the PNs of the subiculum.

## Discussion

*Ube3a* is known to be an important protein with critical role in maintaining the hippocampal long-term potentiation, dendritic spine density (van Woerden et al., 2007; Dindot et al., 2008; Yashiro et al., 2009), behavior (Jiang et al., 1998; Kaphzan et al., 2011, 2013; Koyavski et al., 2019), and various cell signaling pathways (Margolis et al., 2010; Filonova et al., 2014; Tomaić and Banks, 2015). Numerous studies have reported the effects of the overall loss of *Ube3a* in AS mouse brain (Jiang et al., 1998; Yashiro et al., 2009; Kaphzan et al., 2011; Wallace et al., 2012, 2017; Judson et al., 2016; Gu et al., 2019). Correspondingly, electrophysiological investigations in AS mice have revealed several alterations in brain regions such as the primary visual cortex (Wallace et al., 2012; Judson et al., 2016), medial prefrontal cortex (Rotaru et al., 2018), hippocampal CA1 (Jiang et al., 1998; Kaphzan et al., 2011; Rayi et al., 2019, 2020), medial nucleus of the trapezoid body (Wang et al., 2018), striatum (Hayrapetyan et al., 2014), and cerebellum (Bruinsma et al., 2015).

Previous studies in AS model mice showed that the hippocampal region is prominently affected, and many of the hippocampal-dependent behavioral phenotypes are robustly impaired in AS mice (Kaphzan et al., 2011, 2013; Koyavski et al., 2019; Rayi et al., 2019). The subiculum, an adjacent region to the hippocampal CA1 is a major output station relaying information from the CA1 to the cortical and sub-cortical brain regions (Witter et al., 1989; Amaral, 1993). In spite of the numerous electrophysiological studies in different brain regions mentioned above, subicular neurons in AS mice were never investigated. Hence, the purpose of this study was to characterize the subicular PNs using whole-cell electrophysiology in an AS mouse model (Figure 1).

Upon random patching *ex vivo*, we found three distinct neuronal classes in the dorsal subiculum which we classified based on their firing patterns in response to the depolarizing somatic current injections: (1) RF neurons with repetitive single APs, (2) WB neurons with one burst followed by a train of single APs, and (3) SB neurons with two bursts or more followed by repetitive single spikes (Figures 1D–G). Consistent with several studies, we found variable proportions of these neurons in the WT mice (Figure 1H; Taube, 1993; Greene and Totterdell, 1997; Staff et al., 2000). Further, in line with these studies, majority of the subicular neurons in WT mice were intrinsically bursting in nature (WB and SB combined) compared to the RF neurons (Figure 1H). Additionally, we found a similar trend in the proportion of intrinsically bursting neurons in AS mice (Figure 1I). However, we did not observe any significant differences in the overall distribution of different populations between AS and their WT controls (Figures 1H,I). Taken together, these findings reiterate that the bursting PNs make up most of the subicular region. A recent study investigated functional differences among the sparsely bursting and dominantly bursting subicular neurons and reported that the sparsely bursting cells fire less but transfer more spatial information than dominantly bursting neurons and encode spatial information better than single APs (Simonnet and Brecht, 2019). Consistent with this study, we also observed a lower firing rate in WB neurons compared to the SB neurons of WT mice (Supplementary Figure 1).

Alterations in the intrinsic membrane properties of the CA1 PNs have been previously reported in a mouse model of AS (Kaphzan et al., 2011, 2013). Recently, we reported an increased firing frequency of the CA1 PNs in AS mice (Rayi et al., 2019, 2020). Interestingly, in our present study, we observed a reduction in the firing rates of both RF and SB subicular neurons of AS mice (Figures 2A–C, 4A–C), suggesting that the PNs though from adjacent regions (CA1 and subiculum) entail opposite firing characteristics in AS. These regional alterations in spike output could be due to the homeostatic mechanism that occurs to stabilize the hippocampal circuit function by dynamically adapting the neuronal output of the subicular PNs in response to the hyperexcitability in the adjacent CA1 PNs (Turrigiano and Nelson, 2004). The reduction in firing rate could be due to mechanisms such as alterations in ion channel conductance. Specifically, the ion channels that can underlie the reduction in the firing rates, and the reduced RMP and input resistance are most probably the potassium channels. For instance, a background potassium leak by low-threshold voltage-activated potassium currents can explain a reduction in the firing rate in RF and SB neurons (Dagostin et al., 2015). Similarly, other potassium channels such as Kir or K2P channels could also enhance background potassium leak. Such a leak can cause increased persistent potassium efflux near RMP thereby affecting the RMP, the input resistance, and gain modulation of a neuron. A moderate modulation of these channels could explain why the firing frequency is reduced in intermediate current injections in RF neurons and dissipate in higher current injections (Figure 2B), when high threshold voltage-dependent potassium channels dominate and reduce the contribution of the low-voltage or the non-voltage dependent potassium leak channels. When these leak currents are large they would lower the firing rate even in the higher current injections as observed in the SB neurons (Figure 4B). The effects of the reduced input resistance and RMP due to leak currents are also reflected by the increase in the rheobase of the SB neurons in AS mice (Figure 4H). Alternatively, the reduced excitability in subicular RF and SB PNs of AS mice could be also due to alterations in the α1-NaKA expression levels. It is known that the hippocampal CA1 region in AS mice have augmented α1-NaKA levels which result in hyperpolarized RMP and increased excitability (Kaphzan et al., 2011, 2013). Therefore, given the hyperpolarized RMP in both RF and SB neurons in our study (Figures 2F, 4F), similar to the CA1 PNs, it is plausible that the α1-NaKA levels are altered in these subicular PNs in AS mice. However, α1-NaKA expression levels in the subiculum were not investigated in the herein study and needs to be explored further.

While analyzing the intrinsic membrane properties in the different classes of subicular neurons, we found distinct alterations in various neuronal parameters. Contrary to the increased AP amplitude in CA1 PNs of AS mice, we found no differences in the AP amplitude in all subtypes of subicular PNs (Figures 2I–4I). These results suggest that unlike the increased expression of voltage-gated sodium channels Na_V_1.6 in the CA1 PNs of AS mice (Kaphzan et al., 2011, 2013), these channels might be unaltered in the subicular PNs. Another interesting finding was that the input resistance in AS mice was significantly lower only in RF neurons with a trend (*p* = 0.06) toward reduction in SB neurons (Figures 2D, 4D and Supplementary Tables 1, 3), which accompanied the reduction in the excitability in both RF and SB neurons (Figures 2B, 4B). However, the input resistance was not different in the WB neurons of AS mice (Figure 3D), which coincided with the unaltered excitability in these neurons (Figure 3B). Interestingly, we found a reduction in mAHP only in the WB and SB neurons but not RF neurons of AS mice (Figures 3E, 4E), implying the role of calcium-activated potassium channels only in the bursting neurons of these mice (Gulledge et al., 2013; Chen et al., 2014; Tiwari et al., 2018). To this extent, a reduction in mAHP and altered calcium signaling have been previously reported in the adjacent CA1 PNs of AS mice (Rayi et al., 2019, 2020). It is also noteworthy that the ISI ratio is significantly altered only in the SB neurons but not RF and WB neurons (Figures 2K–4K), signifying that the SB neurons in the AS mice have low instantaneous firing rates. Altogether, these alterations in the intrinsic membrane properties of RF, WB, and SB subicular PNs suggest differential expression or dysregulated activity of various ion channels in AS mice (Mattia et al., 1993; Jung et al., 2001; Joksimovic et al., 2017; Panov and Kaphzan, 2021).

The loss of *Ube3a* is known to alter the morphology of axons in AS mice (Judson et al., 2017). Correspondingly, previous studies in AS mice have reported alterations in the spontaneous inhibitory and excitatory synaptic currents in the layer 5 PNs of the prefrontal cortex (Rotaru et al., 2018). Given the heterogeneity in the neuronal population of subiculum, we investigated the synaptic properties of the excitatory inputs to the distinct classes of neurons in AS and WT mice. We found that the RF and WB neurons showed no alterations either in their cumulative distributions of amplitudes and inter-event intervals or the average amplitudes and frequencies between AS and WT controls (Figures 5A–F). It is known that the subiculum receives monosynaptic excitatory input from the CA1 region of the hippocampus (Gigg et al., 2000; O’Mara et al., 2001). However, this input is not exclusive, as subiculum also receives direct input from the entorhinal cortex (Gigg et al., 2000). Therefore, these inputs from CA1 and the entorhinal cortex converge on to the different neuronal populations of the subiculum. Given our results, it is reasonable that the innervations from CA1 and the entorhinal cortex on to RF and WB subicular neurons could be similar in both AS and WT mice. Remarkably, we observed a significant rightward shift in the cumulative frequency curves of amplitudes but not inter-event intervals in SB neurons of AS mice (Figures 5G–I). Nevertheless, the average amplitudes and frequencies were not different between the genotypes (Insets; Figures 5H,I). Altogether, our results suggest that there are no overall changes in the inputs to the different neuronal sub-populations of the subiculum in both AS and WT mice. Further, it is noteworthy that the major alterations in the intrinsic membrane properties were observed mostly in the subicular SB neurons of the AS mice.

Overall, our findings shed light for the first time on the altered intrinsic properties of different types of subicular PNs in AS model mice. Since subiculum plays a critical role in memory processing and spatial encoding, and many neuropsychiatric disorders such as schizophrenia, epilepsy, and Alzheimer’s disease involve impaired activity of the subiculum (Hyman et al., 1990; Carlesimo et al., 2015; Lindberg et al., 2017; Lieberman et al., 2018; Nakahara et al., 2018), it is imperative to understand this region in AS further. Given that AS entails many hippocampal-dependent memory deficits (Jiang et al., 1998; Kaphzan et al., 2011, 2013; Rayi et al., 2019), the electrophysiological characterization of the subicular PNs in these mice is an important step in understanding the pathophysiology of AS. Finally, our data prompts further investigation to elucidate the mechanisms behind the electrophysiological alterations in the subicular PNs of AS mice.

## Data Availability Statement

The original contributions presented in the study are included in the article/Supplementary Material, further inquiries can be directed to the corresponding author.

## Ethics Statement

The animal study was reviewed and approved by University of Haifa Institutional Ethics Committee.

## Author Contributions

PRR performed all the experiments. PRR and HK designed and analyzed the electrophysiology data, conceptualized all the experiments, and wrote the manuscript. HK obtained the funding for the study. Both authors contributed to the article and approved the submitted version.

## Conflict of Interest

The authors declare that the research was conducted in the absence of any commercial or financial relationships that could be construed as a potential conflict of interest.

## Publisher’s Note

All claims expressed in this article are solely those of the authors and do not necessarily represent those of their affiliated organizations, or those of the publisher, the editors and the reviewers. Any product that may be evaluated in this article, or claim that may be made by its manufacturer, is not guaranteed or endorsed by the publisher.
