# Supplementary material for: Electrophysiological Characterization of Regular and Burst Firing Pyramidal Neurons of the Dorsal Subiculum in an Angelman Syndrome Mouse Model
Source: Front Cell Neurosci. 2021 Aug 26;15:670998. doi: 10.3389/fncel.2021.670998 (PMC8427506; doi:10.3389/fncel.2021.670998)
Supplement: Supplementary file 1 [file Data_Sheet_1.docx]

**Electrophysiological characterization of regular and burst firing pyramidal neurons of the dorsal subiculum in an Angelman syndrome mouse model**

Prudhvi Raj Rayi, M.Sc.^1^, Hanoch Kaphzan, M.D., Ph.D., M.P.H.^1^*****

^1^Sagol Department of Neurobiology, The Integrated Brain and Behavior Research Center, University of Haifa, Haifa, 3498838, Israel

***Corresponding author:**

Hanoch Kaphzan

Sagol Department of Neurobiology, University of Haifa

199 Abba Khoushy Ave., Mount Carmel

Haifa 3498838, Israel

Email: [hkaphzan@univ.haifa.ac.il](mailto:hkaphzan@univ.haifa.ac.il)

Phone: 972-53-523008

Fax: 972-4-8240339

**Supplementary Figures and Tables**

**
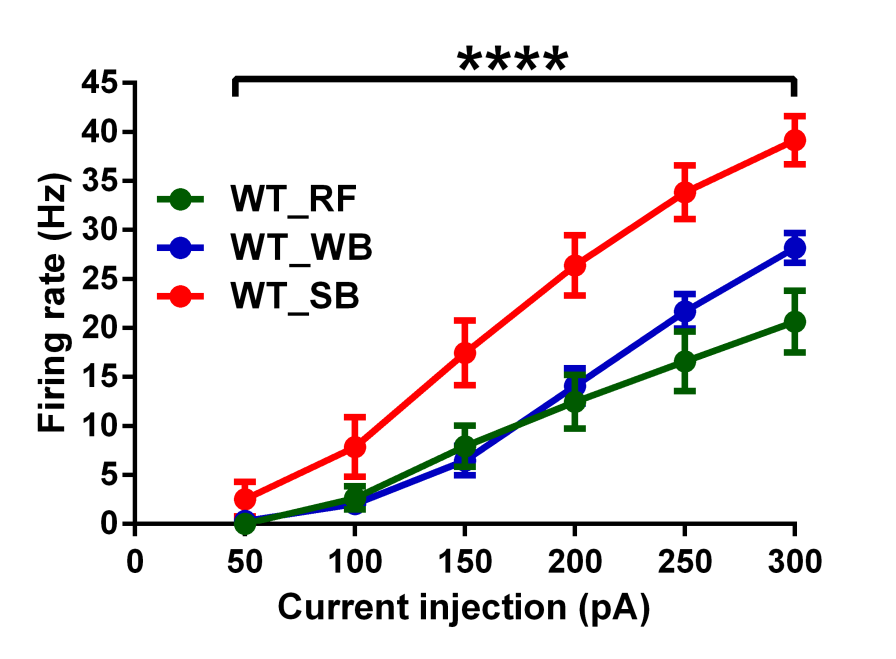
**

**Supplementary Figure 1. Subicular SB PNs of the WT mice show an increased firing rate.** Firing response curves of all subicular PN populations of WT mice summarized according to the current injection steps ranging from 50–300 pA. SB neurons of WT mice exhibit a higher firing frequency compared to the WB and RF neuronal population [F_(2,63)_  =  8.50, *p*  =  0.0005 for main effect of neuronal subtype; F_(10,315)_  =  7.59, *p*  <  0.0001 for interaction of population subtype and current injection in two-way RM ANOVA]. For RF neurons: WT, n = 17 RF neurons, N = 10 mice; AS, n = 16 RF neurons, N = 7 mice. For WB neurons: WT, n = 35 WB neurons, N = 10 mice; AS, n = 40 WB neurons, N = 13 mice. For SB neurons: WT, n = 13 SB neurons, N = 8 mice; AS, n = 10 SB neurons, N = 7 mice. Data are represented as mean ± SEM. *****p* < 0.0001.

**Table 1. Intrinsic properties of regular firing (RF) subicular neurons from WT and AS mice**

| Parameter | RF | | *p*-value |
| --- | --- | --- | --- |
|  | **WT=17 cells** | **AS=16 cells** |  |
| Firing rate 100 pA, Hz | 2.64 ± 1.18 | 0.0 ± 0.0 | *0.04** |
| Firing rate 150 pA, Hz | 7.94 ± 2.09 | 1.37 ± 0.68 | *0.006*** |
| Firing rate 200 pA, Hz | 12.47 ± 2.74 | 5.56 ± 1.71 | *0.04** |
| Firing rate 250 pA, Hz | 16.59 ± 3.03 | 11.50 ± 2.64 | 0.21 |
| Input resistance, MΩ | 81.41 ± 7.91 | 61.27 ± 4.32 | *0.03** |
| mAHP, mV | -5.24 ± 0.73 | -3.59 ± 0.47 | 0.07 |
| RMP, mV | -69.71 ± 0.87 | -72.52 ± 0.61 | *0.01** |
| Threshold potential, mV | -50.66 ± 1.50 | -53.47 ± 1.65 | 0.22 |
| Rheobase, pA | 514.7 ± 48.39 | 490.0 ± 40.53 | 0.70 |
| Amplitude, mV | 90.81 ± 2.39 | 91.58 ± 2.47 | 0.82 |
| Half-width, ms | 1.08 ± 0.05 | 1.05 ± 0.03 | 0.60 |
| ISI ratio | 0.53 ± 0.07 | 0.62 ± 0.06 | 0.39 |

**Table 2. Intrinsic properties of weak bursting (WB) subicular neurons from WT and AS mice**

| Parameter | WB | | *p*-value |
| --- | --- | --- | --- |
|  | **WT=35 cells** | **AS=40 cells** |  |
| Firing rate 100 pA, Hz | 2.09 ± 0.92 | 1.77 ± 0.80 | 0.80 |
| Firing rate 150 pA, Hz | 6.66 ± 1.54 | 6.67 ± 1.48 | 0.99 |
| Firing rate 200 pA, Hz | 14.26 ± 1.85 | 14.28 ± 1.97 | 0.99 |
| Firing rate 250 pA, Hz | 21.83 ± 1.81 | 21.70 ± 2.22 | 0.96 |
| Input resistance, MΩ | 66.53 ± 2.54 | 65.44 ± 2.55 | 0.76 |
| mAHP, mV | -5.08 ± 0.30 | -4.00 ± 0.31 | *0.01** |
| RMP, mV | -69.61 ± 0.49 | -70.59 ± 0.41 | 0.13 |
| Threshold potential, mV | -50.94 ± 0.65 | -52.52 ± 0.73 | 0.11 |
| Rheobase, pA | 466.3 ± 25.29 | 480.5 ± 31.80 | 0.73 |
| Amplitude, mV | 88.36 ± 1.23 | 88.15 ± 1.32 | 0.91 |
| Half-width, ms | 1.05 ± 0.02 | 1.01 ± 0.01 | 0.08 |
| ISI ratio | 0.18 ± 0.01 | 0.23 ± 0.02 | 0.06 |

**Table 3. Intrinsic properties of strong bursting (SB) subicular neurons from WT and AS mice**

| Parameter | SB | | *p*-value |
| --- | --- | --- | --- |
|  | **WT=13 cells** | **AS=10 cells** |  |
| Firing rate 100 pA, Hz | 7.85 ± 3.03 | 0.0 ± 0.0 | *0.03** |
| Firing rate 150 pA, Hz | 17.46 ± 3.30 | 2.00 ± 0.88 | *0.0006**** |
| Firing rate 200 pA, Hz | 26.38 ± 3.06 | 9.30 ± 2.30 | *0.0004**** |
| Firing rate 250 pA, Hz | 33.85 ± 2.74 | 19.20 ± 2.76 | *0.001*** |
| Input resistance, MΩ | 75.13 ± 3.89 | 63.28 ± 4.70 | 0.06 |
| mAHP, mV | -6.08 ± 0.46 | -4.10 ± 0.65 | *0.02** |
| RMP, mV | -67.98 ± 0.71 | -70.47 ± 0.75 | *0.03** |
| Threshold potential, mV | -52.19 ± 1.10 | -49.66 ± 2.60 | 0.34 |
| Rheobase, pA | 344.6 ± 40.99 | 492.0 ± 41.84 | *0.02** |
| Amplitude, mV | 88.52 ± 2.16 | 87.00 ± 3.31 | 0.69 |
| Half-width, ms | 1.08 ± 0.02 | 1.06 ± 0.04 | 0.69 |
| ISI ratio | 0.22 ± 0.01 | 0.15 ± 0.01 | *0.0007**** |
